# Numerical Simulation of Flow and Temperature Fields in a Deep Stratified Reservoir Using Water-Separating Curtain

**DOI:** 10.3390/ijerph16245143

**Published:** 2019-12-16

**Authors:** Lifang Zhang, Jianmin Zhang, Yong Peng, Jiangyang Pan, Zhongxian Peng

**Affiliations:** 1 State Key Laboratory of Hydraulics and Mountain River Engineering, Sichuan University, Chengdu 610065, China; 2017223060052@stu.scu.edu.cn (L.Z.); zhangjianmin@scu.edu.cn (J.Z.); 2 Power China Zhongnan Engineering Corporation Limited, Changsha 410014, China; pjy18380492978@126.com (J.P.); zxpeng1972@163.com (Z.P.)

**Keywords:** standard *k-ε* turbulence, Boussinesq approximation, density-temperature function, water-separating curtain, stratified reservoir

## Abstract

In this work, the flow and temperature fields of a thermally stratified reservoir under different settings of a water-separating curtain are simulated by using the standard *k-ε* turbulence model. In the simulation, two different equations of state including Boussinesq approximation and the density-temperature function have been used and compared. This study shows that Boussinesq approximation is more time-saving, and the density-temperature function has higher computational accuracy. Thus, the standard *k-ε* turbulence model with two equations of state is applied to study the effect of adding a water-separating curtain in the stratified reservoir on the Discharged Water Temperature (DWT). It is found that adding the Water-Separating Curtain (WSC) can effectively increase the discharged water temperature. Moreover, the different arrangements of WSC have obvious effects on the discharged water temperature. For example, the increased temperature by adding a WSC with full sealing is 1 °C higher than that by using the WSC with a bottom opening height of 2 m. However, the maximum pressure difference acting on the WSC for the former WSC is 100 Pa higher than that for the latter WSC. In addition, this study shows that the different equations of state have little effect on the simulation results. Considering the calculation efficiency, equations of state using the Boussinesq approximation can be recommended to save the calculation time.

## 1. Introduction

The construction and operation of large and deep reservoirs have changed the surrounding ecological environment, especially leading to the change of water temperature distribution. The reservoir has formed the thermal stratification of water temperature after it was established [1]. Due to the requirement of power generation efficiency, the inlet of a hydropower station is often located at a low elevation. In the case of stratified water temperature, the discharged water temperature (DWT) is lower in spring and summer than the natural water temperature. That may affect the normal growth of crops and the reproduction of fish, and then cause the reduction or even extinction of crop and fish resources [2,3]. Therefore, the change of water temperature must be regarded as an important index of water environment impact assessment. The water-separating curtain (WSC) can block water in the bottom or top side in order to alleviate the thermal stratification [4,5,6]. It is of great significance to study the water temperature distribution and DWT after WSC is constructed.

In the early 1960s, Orlob and Selna [7] from Water Resources Engineering and Harleman [8] and Huber et al. [9] from Massachusetts Institute of Technology independently developed two vertical, one-dimensional water temperature models, which were well verified and laid a foundation for the establishment of a water temperature model later. A vertical two-dimensional software of modeling the water quality and temperature model named CE-QUAL-W2 was developed by the Water-ways Experiment Station, US Army Corps of Engineers [10]. Bartholow et al. [11] used the prototype observation data of the Shasta reservoir to verify the validity and accuracy of this model. Considering the complex terrain conditions in water conservancy projects, more achievements have been made in the development and application of the three-dimensional hydrodynamic and temperature model such as RMA10 [12], EFDC [13], ECLOM [14] and FLUENT [15], etc. In the above software, the equation of state is needed to consider the relationship between density and temperature. At present, there are two methods of considering this relation: One is to use Boussinesq approximation, which only sets the density to change linearly with temperature in the buoyancy term of the momentum equation while it sets density as a constant in other governing equations, leading to rapid convergence; the other (density-temperature function) is to set the density as a complex polynomial function about temperature in all governing equations to accurately reflect the change of density with temperature. However, the accuracy and computational efficiency of these two methods applied in a deep stratified reservoir are still lacking of relevant research.

In this paper, the laminar model and standard *k-ε* turbulence model with two equations of state (Boussinesq approximation and density-temperature function) will be applied to study the flow and temperature fields. Firstly, the numerical models will be applied to experiments by Johnson (1980) [16]. Based on the simulated results, the longitudinal velocity distribution and the outlet water temperature change will be analyzed, and the differences of calculation efficiency between the two methods are compared. He et al. built a 3-D hydro-thermal-tracer model to study the effect of vertical temperature distribution, water level and flow rate on DWT with no WSC [17]. He et al. also investigated the thermal structure and thrust force on different bottom WSCs and a top WSC [18,19]. This study applying a standard *k-ε* turbulence model to Sanbanxi Reservoir investigates the flow and temperature fields with a water-separating curtain different from the above settings, and the difference of the simulated results using the two equations of state will be studied in detail.

## 2. Mathematical Model and Verification

### 2.1. Governing Equations

Laminar fluid flows are well ordered, where layers of fluid slide past each other. Turbulent flow is a flow regime in fluid dynamics characterized by chaotic changes in pressure and flow velocity. Turbulence leads to an increase in the mixing of momentum and species in the fluid [20]. Almost all CFD simulations of turbulent flows use turbulence models like the *k-ε* model [21]. Turbulence closure models are constitutive relationships between difficult to compute quantities like the Reynolds stress and quantities that are simpler to compute. The *k-ε* model uses the concept of an eddy or turbulent viscosity to derive the Reynolds stress tensor from the turbulent kinetic energy. As turbulence models are simplified relationships, they are approximate and inexact. In complex turbulent flows, their predictions can be quite unsatisfactory. In expanding channels like those investigated in this paper, flow separation occurs in the expansion section. Turbulence models have inexact predictions of the location and the extent of the separation bubble for these cases [22]. This adds a degree of uncertainty to the results of this paper.

The laminar model and standard *k-ε* turbulence model [21] are used in the present study. ANSYS 16.0 (ANSYS^®^, Canonsburg, PA, USA) is used to perform the simulations with the finite volume method. For the laminar model, the governing equations are as follows:

Continuity equation:

(1) $$\frac{\partial \rho }{\partial t}+\frac{\partial \rho u_{i}}{\partial x_{i}}=0$$

Momentum equation:

(2) $$\frac{\partial (\rho u_{i})}{\partial t}+u_{j}\frac{\partial (\rho u_{i})}{\partial x_{j}}=-\rho \frac{\partial p}{\partial x_{i}}+\frac{\partial }{\partial x_{j}}(\mu \frac{\partial u_{i}}{\partial x_{j}})+\rho g_{i}$$

Energy equation:

(3) $$\frac{\partial (\rho T)}{\partial t}+\frac{\partial }{\partial x_{i}}(\rho u_{i}T)=\frac{\partial }{\partial x_{j}}(\frac{\lambda }{C_{p}}\frac{\partial T}{\partial u_{i}})$$

where *t* is time; *p* is pressure; *x* is the Cartesian coordinates; *ρ* is the density of water; *u* is the velocity component; *g* is the acceleration of gravity component; *μ* is the dynamic viscosity; *T* is the water temperature; *λ* is the heat transfer coefficient; *C_p_* is the specific heat of water.

For the standard *k-ε* turbulence model, the continuity equation is Equation (1), and the energy equation is Equation (3). The other governing equations are as follows:

Momentum equation:

(4) $$\frac{\partial (\rho u_{i})}{\partial t}+u_{j}\frac{\partial (\rho u_{i})}{\partial x_{j}}=-\rho \frac{\partial p}{\partial x_{i}}+\frac{\partial }{\partial x_{j}}(\mu \frac{\partial u_{i}}{\partial x_{j}})+\frac{\partial }{\partial x_{j}}[\mu_{t}(\frac{\partial u_{i}}{\partial x_{j}}+\frac{\partial u_{j}}{\partial x_{i}})-\frac{2}{3}(\rho k+\mu_{t}\frac{\partial u_{i}}{\partial x_{i}})\delta_{ij}]+\rho g_{i}$$

*k* equation:

(5) $$\frac{\partial (\rho k)}{\partial t}+\frac{\partial (\rho u_{i}k)}{\partial x_{i}}=\frac{\partial }{\partial x_{j}}\left[(\mu +\frac{\mu_{t}}{\sigma_{k}})\frac{\partial k}{\partial x_{j}}\right]+G_{k}-\rho \varepsilon$$

*ε* equation:

(6) $$\frac{\partial (\rho \varepsilon )}{\partial t}+\frac{\partial (\rho u_{i}\varepsilon )}{\partial x_{i}}=\frac{\partial }{\partial x_{i}}\left[(\mu +\frac{\mu_{t}}{\sigma_{\varepsilon }})\frac{\partial \varepsilon }{\partial x_{i}}\right]+C_{1\varepsilon }\frac{\varepsilon }{k}G_{k}-C_{2\varepsilon }\rho \frac{\varepsilon^{2}}{k}$$

Here,

(7) $$\mu_{t}=\rho C_{\mu }\frac{k^{2}}{\varepsilon }$$

(8) $$G_{k}=\mu_{t}(\frac{\partial u_{i}}{\partial x_{j}}+\frac{\partial u_{j}}{\partial x_{i}})\frac{\partial u_{i}}{\partial x_{j}}$$

*G_k_* represents the generation of turbulence kinetic energy due to the mean velocity gradients; *μ_t_* is the turbulent viscosity which can be deduced for the turbulence intensity *k* and energy dissipation rate *ε*. *σ_k_* = 1.0 and *σ_ε_* = 1.3 are the turbulent Prandtl numbers for *k* and *ε*; *C_μ_* = 0.09, *C*_1*ε*_ = 1.44 and *C*_2*ε*_ = 1.92 are empirical constants. These values have been determined from experiments for fundamental turbulent flows, and have been found to work fairly well for a wide range of wall-bounded and free shear flows.

For the equation of state, if Boussinesq approximation is applied, faster convergence can be obtained. This method treats density as constant in all solved equations except for the buoyancy term in the momentum equation. The linear relationship between density and temperature is shown below:

(9) $$\rho =\rho_{0}[1-\beta (T-T_{0})]$$

where *ρ*_0_ is the reference density of the flow, *T*_0_ is the reference temperature and *β* is the thermal expansion coefficient. This approximation is accurate as long as changes in actual density are small. 

**Figure 1:**
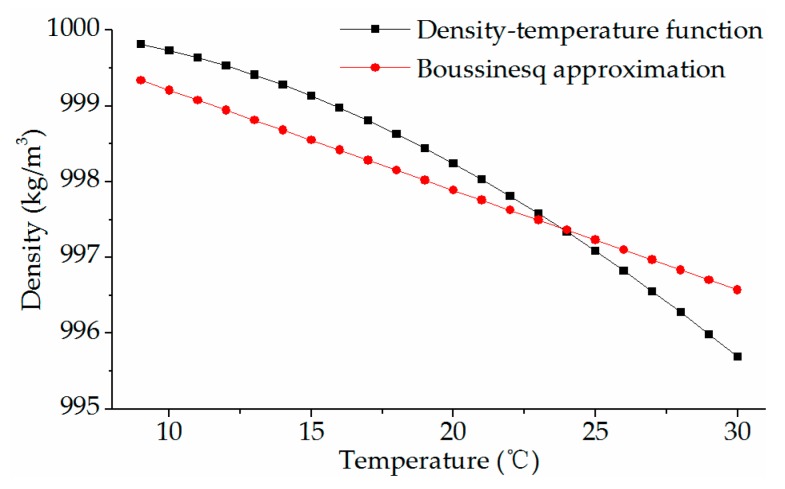
Water density as a function of temperature according to Equations (9) and (10).

Another equation of state is to set density as a polynomial function of temperature [17], which is named as the density–temperature function in this study. The difference in the relationship between density and temperature described by the two methods are plotted in Figure 1. When the temperature varies from 10 °C to 30 °C, the density difference computed by Equations (9) and (10) is less than 1 °C.

(10) $$\begin{array}{l} \rho =(1.02027692\times 10^{-3}+6.67737262\times 10^{-8}\times T-9.05345843\times 10^{-9}\times T^{2}\\ \;+\;8.64372185\times 10^{-11}\times T^{3}-6.42266188\times 10^{-13}\times T^{4}+1.05164434\times 10^{-18}\times T^{7}\\ \;-\;1.04868827\times 10^{-20}\times T^{8})\times 9.8\times 10^{5} \end{array}$$

### 2.2. Model Validation

**Figure 2:**
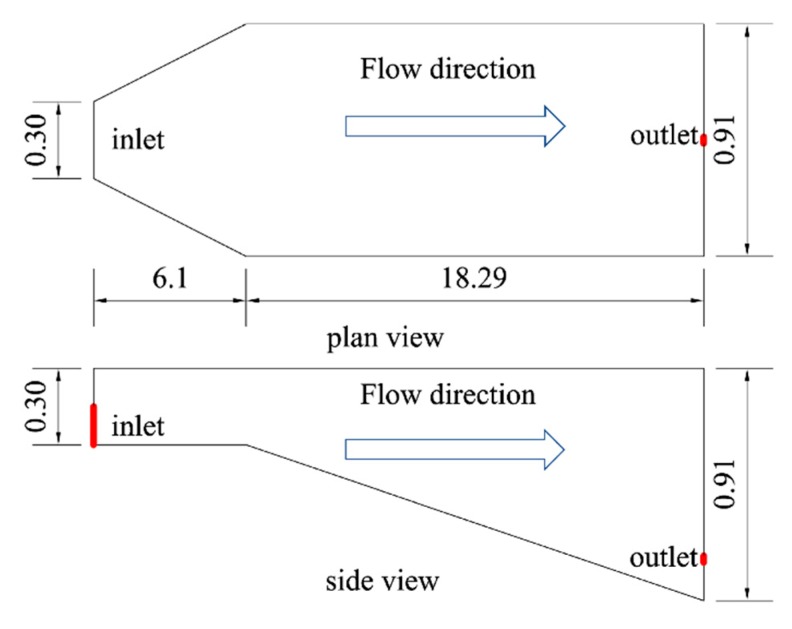
Water tank geometric dimensions for the experiments by Johnson [16]. (Figures are off the scale.).

In this paper, hydrodynamic and temperature models including Boussinesq approximation and the density-temperature function are applied to simulate the water tank experiments conducted by Johnson (1980) [16] from the U.S. Army Engineering Company. The simulation results are compared with the measured data and relevant literature data to verify the validity of the used models. The simulated layout can be seen in Figure 2. The reservoir is 24.39 m long and its depth gradually changes from 0.3 m at the inlet to 0.91 m at the outlet. The change of cross-section shape can be divided into two sections. The first section is 6.1 m long, with a constant height, but the width gradually increases from 0.3 m to 0.91 m. The second section is 18.29 m long, with a constant width, but the height gradually increases from 0.3 m to 0.91 m.

The reservoir is firstly filled with water of 21.44 °C, and then water with 16.67 °C is introduced into the reservoir from the lower part of the inlet section (0.15 m × 0.3 m). The outlet is a hole with 2.54 cm diameter located above 0.15 m from the bottom. Both of the discharges at the inlet and outlet are 0.00063 m^3^/s. The inlet is treated as a velocity-inlet boundary, where the velocity is set to 0.014 m/s and temperature is set to 16.67 °C. The outlet is treated as an outflow boundary. The free surface is treated as an insulated, free-slip rigid lid boundary [23]. The other surfaces of the flume are treated as a no-slip and perfectly insulating wall boundary. After calculation, the inlet Reynolds number is 700 (*Re* = *vR*/*ν*), and the flow was experimentally observed to be laminar and nearly homogeneous across the flume’s width. The laminar flow model and the standard *k-ε* turbulence model are used as shown in Table 1.

**Figure 3:**
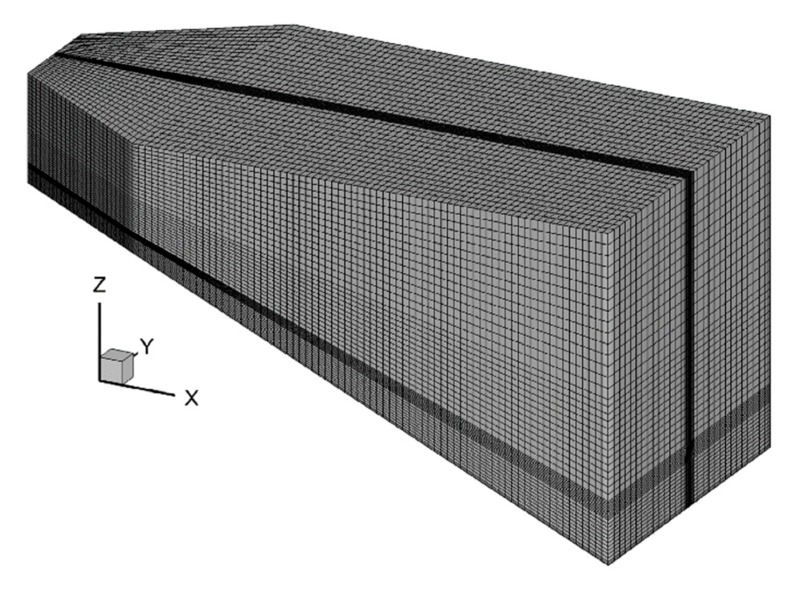
Sketch of the grid generation simulating the experiment by Johnson.

**Figure 4:**
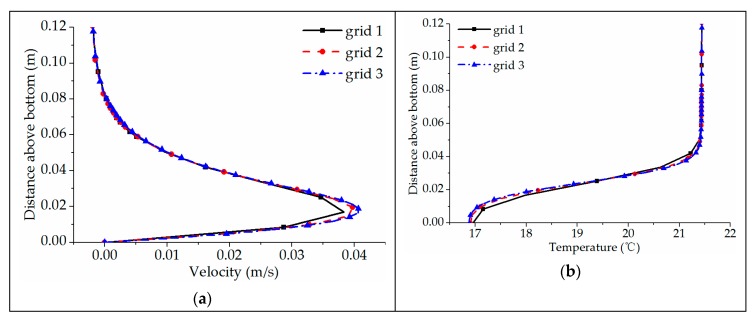
Sensitivity analysis to the vertical grid resolution: (**a**) velocity distribution; (**b**) temperature distribution.

The mesh of the numerical model is configured as a structural grid, as shown in Figure 3. The maximum grid size along the X, Y and Z direction is 0.25 m, 0.05 m and 0.03 m, respectively. Grid refinement is carried out near the bottom and outlet of the flume. It is important to test the uncertainty in the numerical results with different grid sizes. The total grid number is set as approximately 0.11 million (grid 1), 0.21 million (grid 2) and 0.3 million (grid 3), according to different vertical grid resolutions. In the simulation of case A2, the longitudinal velocity and water temperature are obtained on the perpendicular bisector of the cross-section located 10 m away from the entrance at *t* = 30 min. It can be seen from Figure 4 that spatial discretization is fine enough at grid 2. Considering the calculation accuracy and time, the final grid number in this study was taken as 0.21 million.

**Figure 5:**
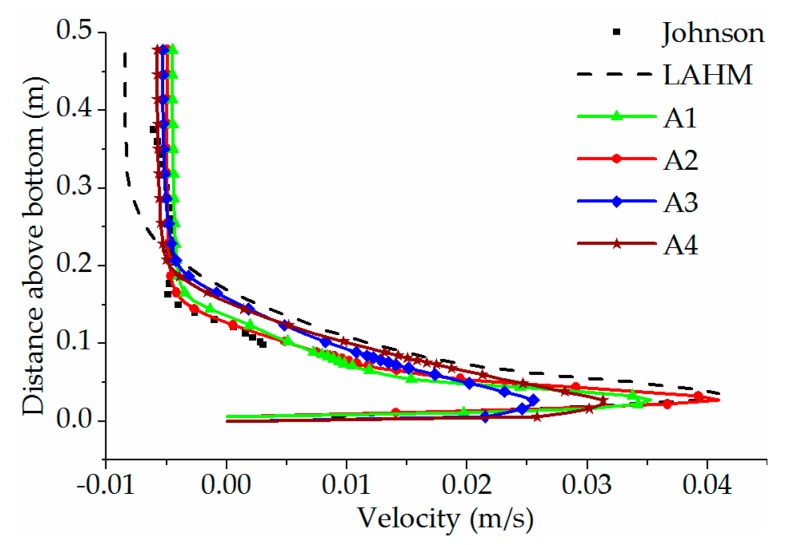
The longitudinal velocity distribution on the perpendicular bisector of the cross-section located 11.43 m away from the entrance at *t* = 11 min.

Figure 5 compares the longitudinal velocity distribution on the perpendicular bisector of the cross-section located 11.43 m away from the entrance at *t* = 11 min. The results show that the movement of the bottom cold-water layer is very fast, while the reverse eddy appears in the upper water body. The reverse velocity of the upper water body agrees well with experimental data. Because the velocity near the bottom plate cannot be measured in experiments, the simulated maximum velocity is compared with those by the LAHM model [23]. LAHM is a laterally averaged hydrodynamics model, which employs a boundary conforming general orthogonal mesh and provides predictions that are superior to those of all other models examined by Johnson [24]. For the bottom phreatic layer, the thickness of the phreatic layer obtained by the standard *k-ε* turbulence model is slightly thicker by 3 cm than experimental data, and the maximum velocity of the cold-water layer is smaller than that by the LAHM model. The thickness of the phreatic layer obtained by the laminar flow model is consistent with the experimental data, and the maximum velocity of the cold-water layer is consistent with that by this LAHM model. Compared with the results by the density–temperature function, the maximum bottom velocity obtained by Boussinesq approximation is smaller.

**Figure 6:**
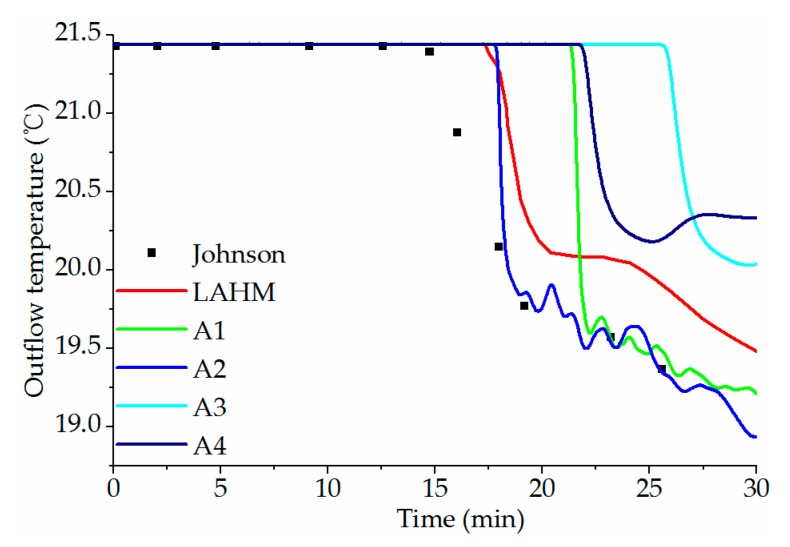
The change of outflow temperature with time.

Figure 6 compares the change of outlet water temperature with time. The experimental results show that the outlet water temperature begins to decrease at *t* = 14.6 min and reduces at 19.36 °C at *t* = 25.6 min. For the laminar flow model using the density–temperature function, although the water temperature at the outlet starts to decline 3.1 min later, and the rate of decline is faster. Then, it drops to 19.35 °C at *t* = 25.6 min and is close to the experiments. 

If the Boussinesq approximation is used, the water temperature drops 6.6 min later, and it drops to 19.48 °C at *t* = 23.5 min. Because the outlet area is small, the flow and temperature fields have three-dimensional characteristics, and the drop of water temperature has slight fluctuation. 

In the simulation results by the standard *k-ε* model, the difference between the results by the two equations of state and the experiment are large, and the time of water temperature drop is later than experiment. The possible reason is that the vertical diffusion and mixing are considered in the standard *k-ε* model, which makes the phreatic layer thick and the longitudinal movement speed is small.

**Figure 7:**
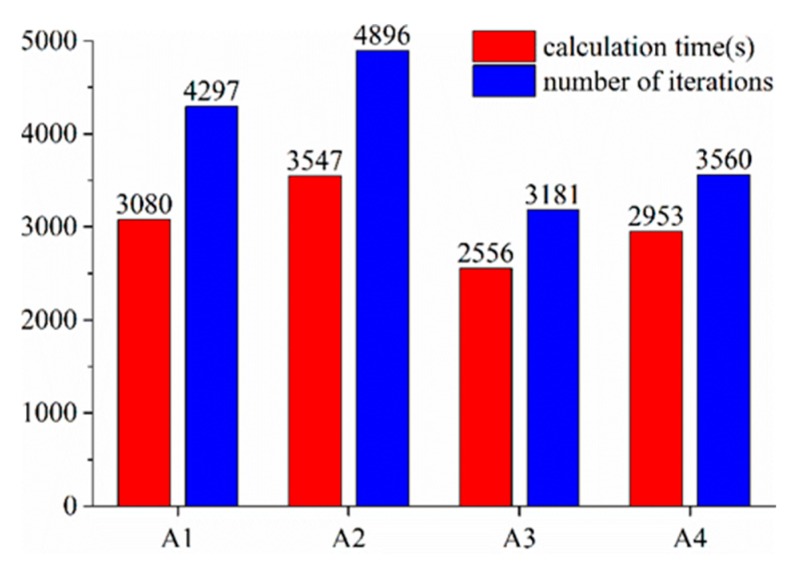
Computational efficiency for different cases.

Figure 7 shows the computational efficiency for different cases. The total grid number of the computing model is 210,800, and the used CPU is Intel^®^ core™ i7-4790 (3.60 GHz). Using the same calculation model, computing time of Boussinesq approximation is shorter, and the number of iteration steps is smaller, while the density–temperature function increases the nonlinearity of the equations, so it needs more computing time.

Overall, the laminar model used in this paper is more accurate and reliable than the LAHM model. The moving velocity of the cold-water layer simulated by the laminar model is consistent with the experiments, while the results by the standard *k-ε* model are relatively backward. Compared with the Boussinesq approximation, the density–temperature function can get better results for the same calculation model, but it needs more computation power.

## 3. Case Study 

The study area is the Sanbanxi Reservoir (26°36′ N, 109′3 E) located in east Guizhou Province, China, and the upstream of Yuanjiang River. After the reservoir was constructed, the annual average maximum water depth exceeded 130 m. Due to seldom flooding, the water temperature in front of the dam was obviously stratified, which resulted in the discharge of low-temperature water and brought adverse effects on fish spawning in the downstream. In order to make fish have suitable water temperature during the spawning period, it is necessary to set WSC to increase the DWT. Because the setting of WSC makes the turbulence of the flow field in the reservoir more violent, it is more accurate to use the standard *k-ε* model to simulate the temperature field and flow field in the reservoir. The stratified water temperature and the effect of the water-separating curtain (WSC) on improving DWT are investigated in detail.

### 3.1. Computational Layout

**Figure 8:**
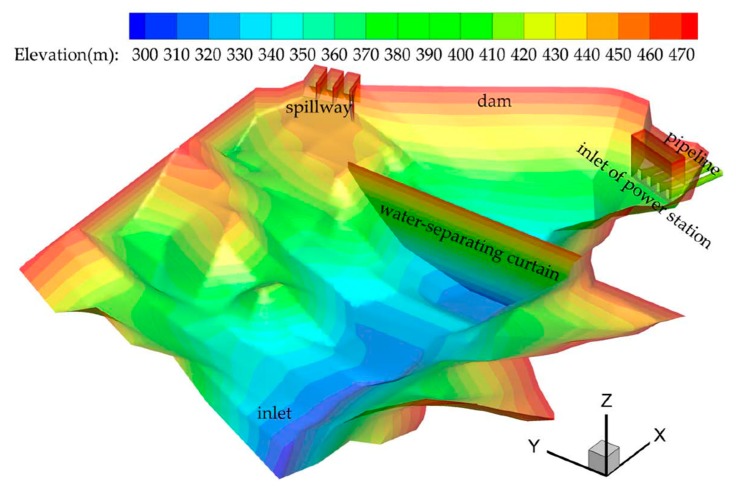
Layout of the computational model.

The computational model layout is shown in Figure 8. According to the layout of the hydropower station project, it includes hydraulic structures, such as the main dam, the spillway, the water intake of the power station and the diversion pipeline. The simulated range includes the reservoir area of 1.0 km in front of the dam and four diversion pipelines of the power station. The free surface elevation is 475 m a.s.l. The WSC is at the location of 250 m in front of the inlet of the power station. The elevation of the inlet of the power station is in the range of 408–424 m a.s.l. 

**Figure 9:**
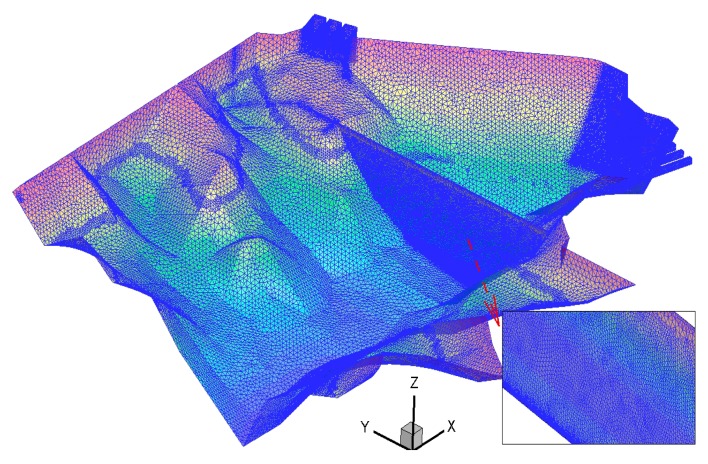
Sketch of the grid generation.

In order to save computational time and simulate the complex terrain, the non-uniform unstructured grid is adopted for grid generation, and refined grids are carried out for key parts. Grid generation is shown in Figure 9.

**Figure 10:**
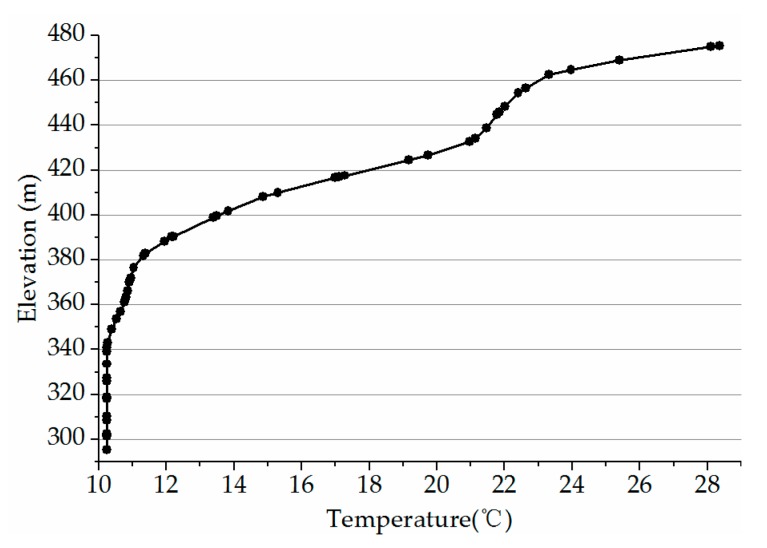
Vertical distribution of inlet and initial water temperature.

For the inlet boundary, the velocity-inlet boundary condition is adopted with a flow rate of 870 m^3^/s, and the inlet water temperature distribution adopts the measured data at the section 1.0 km upstream from the dam. The initial vertical temperature distribution is the same as the inlet, as shown in Figure 10. The discharge of the power station intake is 870 m^3^/s, and the spillway is closed, so the outlet boundary condition is set at the downstream of four pipelines with a flow rate of 870 m^3^/s. Considering that the discharge flow rate is small and water level changes little before the dam, the rigid-lid assumption is adopted to deal with the free surface, which assumes the top is a free-slip wall and the normal velocity gradient is zero [23]. The reservoir bottom is set as the no-slip wall boundary condition. Because the WSC is fixed in the reservoir by anchor and floater, and made of high tenacity polypropylene geotextile with extremely low water permeability, it is simplified as a rigid and adiabatic wall inserted into the reservoir. After referring to the relevant authority and geological conditions, bottom Manning’s coefficient is set as 0.035 s/m^1/3^.

### 3.2. Numerical Simulation

The backwater length of Sanbanxi Reservoir is 120.9 km, with a total storage capacity of 4.094 billion m^3^. The simulation range is only 1 km in front of the dam, and the simulation time is short, so the effect of atmospheric temperature and solar radiation is very small and can be ignored. The top opening height of WSC is 20 m below the free surface. 

As is shown in Table 2, two equations of state are respectively applied to simulate the temperature field with different bottom opening heights (*BOH*) of WSC. *BOH* is the distance from the bottom of WSC to the bottom of the reservoir. 

### 3.3. Results and Discussion

#### 3.3.1. Flow Field and Temperature Field

**Figure 11:**
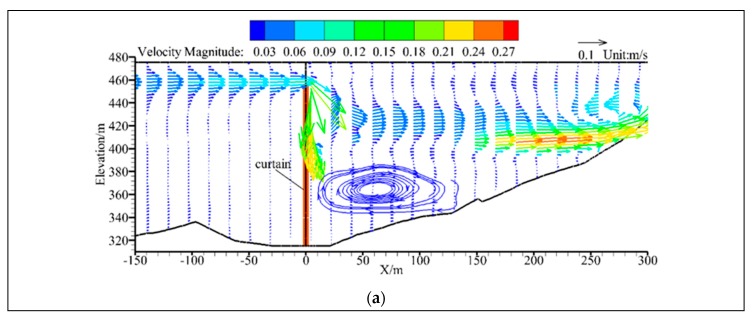

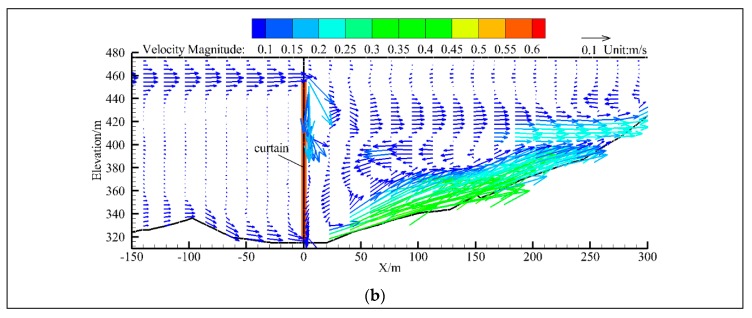
Flow field of Y = 400 m cross-section: (**a**) bottom opening heights (*BOH*) = 0 m; (**b**) *BOH* = 2 m.

Since the simulated flow and temperature fields change very little using the two equations of state, cases B1 and B2 are taken for analysis in this section. The cross-section of Y = 400 m is tangent to the deepest part of the WSC’s axis, and the velocity vector of this cross-section is shown in Figure 11. From Figure 11a, it can be seen that the mainstream in the reservoir area is distributed on the surface layer when the bottom of the WSC is completely sealed, and quickly dives after passing the WSC. The mainstream is distributed in the range of elevation of 400–440 m a.s.l. The maximum flow velocity reaches 0.27 m/s, forming a small flow return vortex at the bottom. On the other hand, it can be seen from Figure 11b that there is also another mainstream distributed on the bottom layer due to the opening of WSC’s bottom, and its flow pattern is similar to jet flow after passing through the WSC. The flow velocity at the bottom opening of WSC reaches a maximum of 0.6 m/s. Then, both of the surface mainstream and the bottom mainstream moves into the inlet of the power station.

**Figure 12:**
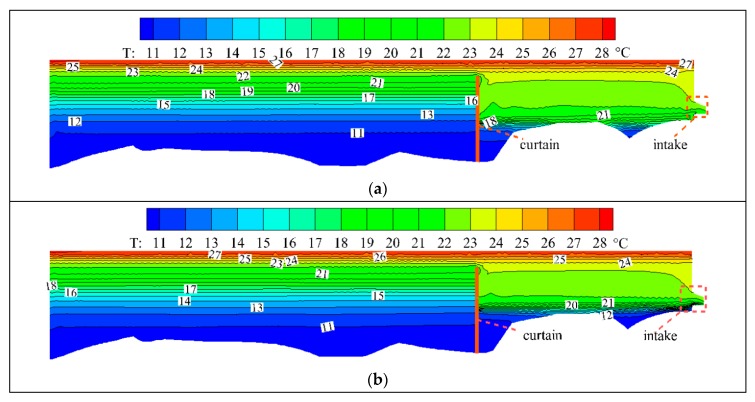
Temperature field of Y = 335 m cross-section: (**a**) *BOH* = 0 m; (**b**) *BOH* = 2 m.

The cross-section of Y = 335 m is tangent to the center point of the inlet of the power station. Figure 12 shows the temperature field distribution of this cross-section. For the temperature field in front of the WSC, the distributions in the two cases are similar. Because the bottom full seal has a stronger blocking effect on the flow in the middle and lower parts of the reservoir, the accumulation effect of low-temperature water is more obvious. For the temperature field behind the WSC, the 23 °C isothermal layer is sucked into the inlet of the power station, and the location of the low-temperature water for the bottom full seal condition is low, most of which is stuck in the bottom of the reservoir area, and cannot be discharged. Affected by the bottom opening, the location of the low-temperature water for the case with *BOH* = 2 m is higher, and it has jet flow pattern, which drives the low-temperature water to discharge into the inlet of the power station. It shows that the opening at the bottom of the WSC has some influence on the DWT at the inlet of the power station due to the influence of the flow field.

#### 3.3.2. Temperature Difference

**Figure 13:**
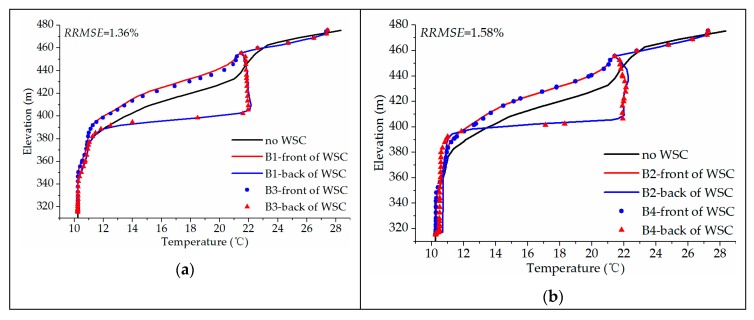
Comparison of vertical water temperature distribution in front and behind the curtain simulated by different equations of state: (**a**) *BOH* = 0 m; (**b**) *BOH* = 2 m.

Figure 13 shows the vertical water temperature distribution in front of and behind the WSC. Due to adding the WSC, the temperature distribution of the original reservoir is obviously changed.

The reason for the water temperature difference between the front of the WSC and behind the WSC, as well as the water temperature at the inlet, is that the water flow in the middle and lower parts cannot be discharged, which causes the accumulation of low-temperature water flow and compresses the temperature change layer. Under the two cases, the water temperature distribution in front of the WSC is similar and the WSC has an obvious blocking effect on the low-temperature water in the bottom layer. For the case with *BOH* = 2 m, the low-temperature water can partially enter the reservoir downstream of the WSC through the opening at the bottom of the WSC, so the blocking effect is slightly weak. Therefore, the water depth of the low-temperature water is slightly smaller than that for the case of the full sealing.

The water temperature distribution behind the WSC is significantly different: for the case of *BOH* = 0 m, from the top of the WSC to an elevation of 400 m a.s.l., the water temperature has a slight upward trend along the water depth direction due to the mixing of high-temperature water at the top and low-temperature water in the middle. Basically, it remains at about 22 °C. After that, it suddenly drops to 11 °C. The water temperature below Z = 390 m both in the front and back of WSC is almost the same, which shows that the water temperature is not disturbed. 

For the case of *BOH* = 2 m, the temperature changes from the elevation of 400 m a.s.l. and decreases rapidly to less than the water temperature in front of the WSE. From elevation of 355 m a.s.l. to the bottom of the WSC, the water temperature behind the WSC is higher than the that in front of the WSC, and the vertical change of the low-temperature water in the bottom is smaller than that in front of the WSC, indicating that the low-temperature water in the bottom is mixed violently under the influence of the bottom return vortex.

Relative Root Mean Square Error (*RRMSE*) is used to compare the errors between the simulated results of the two equations of state. The formula is as follows:

(11) $$RRMSE=\sqrt{\frac{1}{n}\sum_{i=1}^{n}{\left(\frac{y_{i}-u_{i}}{u_{i}}\right)}^{2}}\times 100\%$$

where *RRMSE* is the relative root mean square error, *n* is the total number of all data points, *y_i_* is the simulated result by density-temperature function, *u_i_* is the simulated result by Boussinesq approximation. The *RRMSE* values of the two equations of state are 1.36% and 1.58%, respectively, which shows that the simulation results for the two methods are similar.

**Figure 14:**
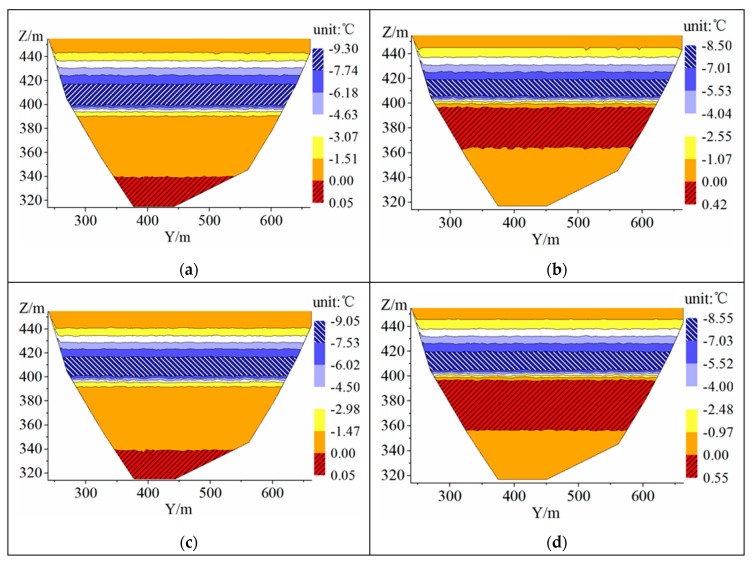
The contour of temperature difference: (**a**) B1; (**b**) B2; (**c**) B3; (**d**) B4.

Figure 14 shows the distribution of the temperature difference between the upstream and the downstream of the WSC under different cases. The temperature difference (Δ*T*) is defined as the upstream temperature of the WSC minus the downstream temperature of the WSC. For the cases of *BOH* = 0 m (B1, B3), when the elevation is higher than 340 m a.s.l., Δ*T* is less than 0 (that is to say, at the same elevation, the water temperature at the upstream of the WSC is lower than that at the downstream of the WSC.). The maximum negative Δ*T* is 9.3 °C and 9.05 °C respectively, which appears near the elevation of 410 m a.s.l. Under the influence of the backflow vortex at the bottom of the WSC, the temperature field behind the WSC is slightly disturbed, and there is a small positive Δ*T* at the bottom of the WSC. Moreover, the maximum positive Δ*T* is 0.05 °C. For the cases of *BOH* = 2 m (B2, B4), the large temperature difference is distributed near an elevation of 415 m a.s.l., and the maximum negative Δ*T* is 8.50 °C and 8.55 °C, respectively. The positive Δ*T* is distributed at an elevation of 360–390 m a.s.l., and that is caused by the moving of low-temperature water at the bottom. Moreover, the moving of low-temperature water at the bottom causes the serious accumulation of low-temperature water behind the WSC, leading to larger positive Δ*T*, up to 0.42 °C and 0.55 °C, respectively.

#### 3.3.3. Pressure Difference

**Figure 15:**
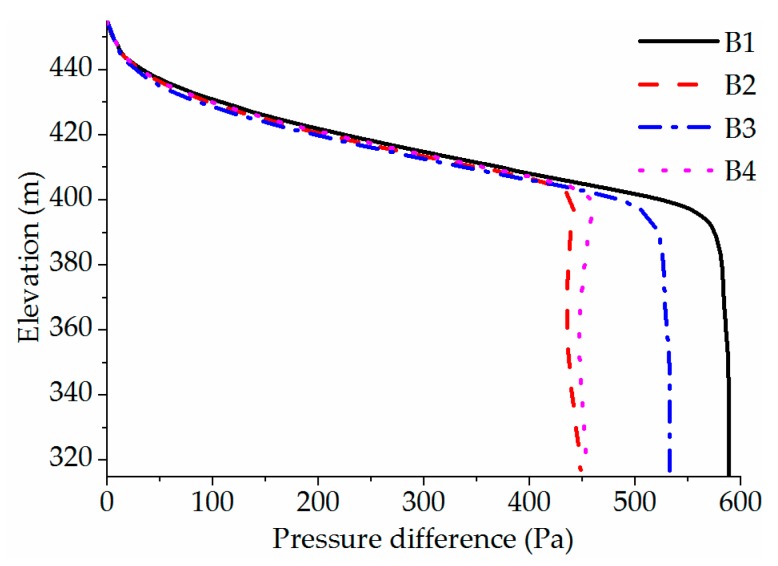
Pressure difference varies with elevation under four cases.

Figure 15 shows how the pressure difference varies with elevation under four cases. The pressure difference (Δ*P*) is defined as the upstream pressure of the WSC minus the downstream pressure. Δ*P* is mainly due to the existence of Δ*T*, resulting in the difference of density along the direction of water depth, which is no longer a fixed value. According to the distribution law of hydrostatic pressure, the pressure on both sides of the same submergence depth below the top of the curtain is different. For the cases of *BOH* = 0 m (B1, B3), at an elevation of 400–440 m a.s.l., Δ*P* increases along the direction of water depth, and the rate of change is fast. The reason is that Δ*T* in this water level range is large. Below the elevation of 340 m a.s.l., Δ*P* slightly reduces, corresponding to the positive Δ*T* phenomenon at the bottom of the WSC. But the variation is small, and the main reason is that the positive Δ*T* is only 0.05 °C. In addition, the maximum Δ*P* is 590 Pa and 534 Pa, respectively. For the cases of *BOH* = 2 m (B2, B4), the distribution of Δ*P* is similar to that of the fully sealed bottom at the elevation above 400 m a.s.l. Whereas, at the elevation of 360–400 m a.s.l., Δ*P* decreases, corresponding to the positive Δ*T* phenomenon of the WSC. It is found that the maximum Δ*P* is 450 Pa and 460 Pa, respectively. According to the above analysis, Δ*T* is the derivative of the Δ*P* curve. Because the area of WSC is almost 40,000 m^2^, the existence of Δ*P* directly affects the thrust force acting on the WSC. Above all, the bottom opening height of the WSC can effectively reduce the thrust force acting on the WSC.

#### 3.3.4. Discharged Water Temperature

The discharged water temperature of the power station is an important index to measure the effect of WSC. Table 3 lists the simulation results for different cases. It can be found that the setting of WSC can effectively increase the DWT. When the bottom of the WSC is fully sealed, it can increase the DWT by about 4 °C, which is better than the case of *BOH* = 2 m. Compared with the simulation results of different equations of state, the difference of water temperature at the outlet is about 0.2 °C, which shows that it has little effect on temperature simulation.

## 4. Conclusions

In this paper, the laminar flow model and standard *k-ε* turbulence model have been used to simulate the flow field and temperature field of thermally stratified flow. Moreover, two different equations of state including Boussinesq approximation and the density–temperature function have been incorporated into the above models and compared in detail. Based on the study, the following conclusions can be drawn as follows:

1. The laminar flow model and standard *k-ε* turbulence model including the Boussinesq approximation and the density–temperature function have been verified by the experiment of Johnson (1980). It is found that the density–temperature function has better accuracy, and the simulation results by laminar flow model combined with the density–temperature function are the most accurate because the flow pattern in the experiments is laminar flow. However, using the density–temperature function equation needs more computing resources and longer computing time.
2. The standard *k-ε* double equation model is applied to the water temperature simulation of the actual reservoir, and the influence of different layouts of WSC on the flow field and temperature field is analyzed. The study shows that setting WSC in the thermally stratified reservoir can effectively increase the discharged water temperature of the power station.
3. Moreover, the different arrangements of water-separating curtain has obvious effects on the discharged water temperature. For example, the increased temperature by adding a WSC with full sealing is 1 °C higher than that by using the WSC with a bottom opening height of 2 m. However, the maximum pressure difference acting on the WSC for the former WSC is 100 Pa higher than that for the latter WSC. When the layout of the WSC is used, both of the temperature rise and the force acting on the WSC need to be considered together.
4. Different equations of state have little effect on the simulation results. Considering the influence of calculation efficiency, Boussinesq approximation can be recommended to study the water temperature distribution of large reservoirs.

## Figures and Tables

**Table 1:** Governing equations used in simulation.

Cases	Calculation Model	Equation of State
A1	Laminar	Boussinesq approximation
A2	Laminar	Density-temperature function
A3	Standard *k-ε*	Boussinesq approximation
A4	Standard *k-ε*	Density-temperature function

**Table 2:** Water-separating curtain (WSC) layout and equation of state.

Cases	Top Opening Height (m)	Bottom Opening Height (m)	Equation of State
B1	20	0	Density-temperature function
B2		2	Density-temperature function
B3		0	Boussinesq approximation
B4		2	Boussinesq approximation

**Table 3:** Discharged water temperature and heating effect of the power station.

Cases	Discharged Water Temperature/°C	Effect of Increasing DWT/°C
No WSC	17.78	/
B1	21.85	4.07
B2	21.09	3.31
B3	21.97	4.19
B4	21.29	3.51
